# Chemical, Mechanical, and Durability Properties of Concrete with Local Mineral Admixtures under Sulfate Environment in Northwest China

**DOI:** 10.3390/ma7053772

**Published:** 2014-05-13

**Authors:** Qingke Nie, Changjun Zhou, Xiang Shu, Qiang He, Baoshan Huang

**Affiliations:** 1 Hebei Research Institute of Construction & Geotechnical Investigation Company Limited, Research Center for Geotechnical Engineering of Shijiazhuang, Shijiazhuang 050031, Hebei, China; E-Mail: nieqingke2001@sina.com; 2 School of Transportation Science and Engineering, Harbin Institute of Technology, Harbin 150090, Heilongjiang, China; E-Mail: czhou5@utk.edu; 3 Department of Civil and Environmental Engineering, University of Tennessee, Knoxville, TN 37996-2010, USA; E-Mails: xshu@utk.edu (X.S.); qhe2@utk.edu (Q.H.)

**Keywords:** concrete, mineral admixtures, sulfate attack, durability, chloride permeability

## Abstract

Over the vast Northwest China, arid desert contains high concentrations of sulfate, chloride, and other chemicals in the ground water, which poses serious challenges to infrastructure construction that routinely utilizes portland cement concrete. Rapid industrialization in the region has been generating huge amounts of mineral admixtures, such as fly ash and slags from energy and metallurgical industries. These industrial by-products would turn into waste materials if not utilized in time. The present study evaluated the suitability of utilizing local mineral admixtures in significant quantities for producing quality concrete mixtures that can withstand the harsh chemical environment without compromising the essential mechanical properties. Comprehensive chemical, mechanical, and durability tests were conducted in the laboratory to characterize the properties of the local cementitious mineral admixtures, cement mortar and portland cement concrete mixtures containing these admixtures. The results from this study indicated that the sulfate resistance of concrete was effectively improved by adding local class F fly ash and slag, or by applying sulfate resistance cement to the mixtures. It is noteworthy that concrete containing local mineral admixtures exhibited much lower permeability (in terms of chloride ion penetration) than ordinary portland cement concrete while retaining the same mechanical properties; whereas concrete mixtures made with sulfate resistance cement had significantly reduced strength and much increased chloride penetration comparing to the other mixtures. Hence, the use of local mineral admixtures in Northwest China in concrete mixtures would be beneficial to the performance of concrete, as well as to the protection of environment.

## 1. Introduction

### 1.1. Background

The Northwest China, including Xinjiang, Gansu, Qinhai, partial Tibet, and partial Inner Mongolia, spans over 3.5 million square kilometers. A significant portion of this area is covered by arid desert, which has similar geographic terrain to that of central Asia where the ancient “Silk Road” passed through. Due to low natural precipitation, saline soils are typical in this arid region. Salts in the soil are mainly from the weathering of minerals or the upward movement of shallow groundwater. Field measurement indicates that the water-soluble sulfate (SO_4_) content is typically 0.7% by mass in the soil and 12,000 ppm in groundwater. According to the American Concrete Institute (ACI), it can be classified as Class II to III (severe to very severe) sulfate exposure conditions. Therefore, portland cement concrete used in this sulfate environment should be specially designed for improved durability.

Portland cement concrete (PCC) may be vulnerable when exposed to environment rich in sulfate ions [1]. Sulfate attack often occurs in arid areas with highly concentrated sulfate in soils. When exposed to ground water, external sulfates in the soil can be dissolved and cause damage to foundations, retaining walls, and other underground concrete structures [2,3]. In addition to sulfate, other chemicals such as chloride ions, when penetrated through concrete, may accelerate the corrosion of steel reinforcement within concrete, and poses serious threat to infrastructures [4].

With the rapid industrialization of the Northwest China, huge amounts of by-products are being generated from various industries in the region. Notably, the coal combustion power plants and metallurgical industries in the Northwest China are the key pillars to support the local industrialization. Taking Xinjiang Urgur Automatous Region (a key province in Northwest China) as an example, in 2012, the automatous region produced 9 million kilowatt-hour of electricity from coal power plants and 2.4 million tons of steel, generating a huge amount of by-products including fly ash, silica fume and blast furnace slag. These mineral admixtures, if not utilized in time, would turn into waste materials and poses serious threat to the environment.

It has been well documented that mineral admixtures or supplementary cementing materials (SCMs) such as fly ash and blast furnace slag may be used in portland cement concrete and improve the durability of concrete, such as sulfate resistance [5–8]. In addition, the production of portland cement generates a huge amount of carbon dioxide. Utilizing SCMs in concrete as a partial substitution of cement is also an effective way to reduce the greenhouse gas emission from concrete products [9,10]. Considering the large scale consumption of concrete in the construction of infrastructures, the economic and environmental benefit of utilizing these local mineral admixtures in concrete would be tremendous.

### 1.2. Previous Studies

Previous studies found a positive relationship between the tri-calcium aluminate (C_3_A) content in portland cement and the expansion of cement mortar and concrete [11]. Field observations in Canada also support this conclusion [12]. The resistance of concrete to sulfate attack can be improved by using sulfate-resisting cement with low C_3_A content [13] or adding supplementary cementitious materials (SCMs) such as fly ash and slag [5].

Many studies have been conducted to evaluate the properties of concrete with SCMs from mechanical [14], chemical, durable perspectives [8,15,16]. Fly ash and slag were found to be able to increase concrete’s strength and sulfate resistance [7,8,17–19]. Fly ash and slag can significantly reduce the sulfate deterioration by reducing the amount of free lime and reactive aluminates for sulfate reaction and reducing concrete permeability.

In addition to improve concrete’s sulfate resistance, fly ash was also found to mitigate alkali-silica reaction (ASR) in concrete [20,21], and decreases the shrinkage of cement paste [22]. Adding SCMs also considerably decrease the chloride permeability of concrete [23]. Liu *et al.* [24] optimized SCM content for sustainable concrete mixtures through evaluation of chloride ion penetration, freeze-thaw resistance, and compressive strength in State of Colorado. According to the test results, the maximum possible cement replacement percentage with the fly ash was 50%.

However, mineral admixtures, due to their variable sources and procedures, vary significantly in chemical compositions as well as interactions with cement [25,26]. Previous studies showed that fly ash from different coal sources in Europe have varying chemical characteristics and concrete strength [26,27]. Due to the size of its scale, as well as lack of understanding to its interactions with cement concrete, it is expedient to investigate the applicability and the effects of Northwest’s local mineral admixtures in portland cement concrete.

## 2. Objective and Scope

The objective of the present study was to determine whether typical local mineral admixtures from Northwest China could be used in significant quantities to produce concrete mixtures that can resist the harsh sulfate environment without compromising the necessary mechanical properties. To achieve this objective, comprehensive laboratory testing was considered to characterize the chemical, mechanical, and durability properties of cementitious materials and concrete. Chemical and thermal analyses were considered to characterize chemical components and C_3_A concentrations of the cementitious materials. To evaluate the mechanical and durability of the cement mortar and concrete, compressive strength, sulfate resistance and chloride penetration tests were conducted.

## 3. Materials

Local coarse aggregates, natural sand and cementitious materials were utilized in this study. Cementitious materials included two types of local portland cements: ordinary portland cement (OPC) and sulfate resisting cement (SRC) with a strength level of 32.5 PMa, two types of local Class F fly ash and two types of local slags, named as S75 and S95. Except for the concretes in the rapid chloride ion permeability test (RCIP), standard graded sand [28] utilized in all other tests in this study. In China, concrete is classified according to the levels of compressive strength. For instance, C20 concrete represents a concrete with at least 20 MPa in its 28-day compressive strength, at a 95% confidence level. In RCIP test, three types of concrete mixtures were considered, including C20, C30 and C40. The proportion of the natural sand in the aggregate for concrete in RCIP test ranged from 50% to 60%.

## 4. Chemical Compositions of Cementitious Materials

Table 1 shows chemical components of the cementitious materials. It can be seen that the SCMs, especially fly ashes, contain much more SiO_2_ but less Al_2_O_3_ than cements. The hydraulic and pozzolanic reactions of SCMs produce calcium silicate hydrate (C-S-H). Also the pozzolanic reaction consumes the porous calcium hydroxide (Ca(OH)_2_), which is prone to sulfate attack. With proper selection and proportions of SCMs, the two reactions mentioned above make the microstructure in the concrete denser than ordinary portland concrete [29], which makes the concrete less permeable. Low Al_2_O_3_ content would also reduce the reactive aluminates in concrete and potentially improve the sulfate resistance. In addition, the use of SCMs decreases the content of C_3_A in cementitious materials, and thus the amount of AFm (C_3_A·CaSO_4_·12H_2_O) in concrete, leading to a reduced risk of sulfate attack.

## 5. Chemical Compounds of Cementitious Materials

According to ASTM 1365 [30], the quantified X-ray diffraction analysis tests were carried out on the cementitious materials. It is found in Table 2 that SRC contains much less C_3_A than OPC, indicating SRC concrete may be more durable than OPC concrete in sulfate environment. Table 3 shows the X-ray diffraction analysis results on SCMs. It can be seen that although S75 and S95 are very similar in their chemical compositions, they have very different chemical compounds, indicating they might behave differently on improving concrete resistance on sulfate attack when added to concrete. Detailed analyses on the results of slags were not given since fly ash rather than slag was popularly used in the construction. Similar chemical compounds were found in the two fly ashes. According to their chemical compounds, pozzolanic reaction will be triggered if they are used to mix concrete. The pozzolanic reaction will consume Ca(OH)_2_ and produce C-S-H, making concrete less permeability and more durable to sulfate and chloride attack.

## 6. Thermal Analysis of Cementitious Mixtures during Hydration

**Figure 1.**
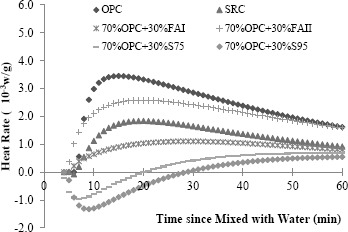
Rate of hydration heat of different cementitious materials.

**Figure 2.**
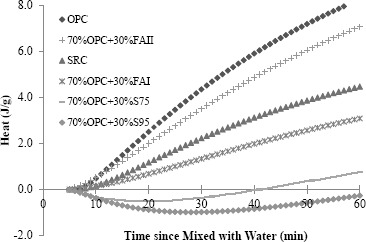
Accumulated hydration heat of different cementitious materials.

Hydration heat of cementitious material is determined by its chemical components. Among the main compounds in cement, C_3_A reacts with water immediately when exposed to water and releases large amount of heat in a very short time. Thus, the content of C_3_A can be estimated by comparing the heat releasing rate of cementitious materials, which intimately relates to the potential sulfate resistance of the cementitious mixture. In accordance with ASTM C 1679 [31], the hydration heat of the cementitious mixtures with different combination of cement and SCMs was measured by an isothermal heat conduction calorimetry as a function of time. The mass of each sample is about 200 g. The water to cementitious material (*w*/cm) ratio was 0.4. Two duplicated samples were prepared and tested. Figure 1 shows the heat rates of different cementitious materials. It can be seen that, ordinary cement has much higher heat rate (a maximum value of 3.3 × 10^−3^ *w*/g), indicating a higher C_3_A content. As SCMs were added into ordinary portland cement, the heat rate was reduced significantly (for instance, a maximum value of 2.4 × 10^−3^ *w*/g with 30% FAII added in), indicating lower C_3_A content. Therefore the sulfate resistance of cementitious materials is expected to be improved. Figure 2 gives the accumulated hydration heat of different cementitious materials. It can be seen with supplementary cementitious materials added, the heat of cementitious materials released in first three days decreased significantly. For instance, after 50 h, the total heat released from OPC is 6.8 J/g, while the total heat released from 70%OPC + 30%FAI mix is only 2.3 J/g.

## 7. Laboratory Tests of Hardened Mixtures

In addition to satisfy specific strength requirement, concrete used in severe sulfate environment should be designed to have sufficient durability. Compressive strength tests of cement mortar were conducted to evaluate the mechanical property of designed concrete. As mentioned above, to increase the sulfate resistance, the concrete should have lower permeability and less active chemical components prone to sulfate corrosion. Sulfate resistance and rapid chloride penetration tests were conducted to evaluate the permeability of concrete.

### 7.1. Compressive Strength of Cement Mortar

In accordance of ASTM C109 [32], cement mortar cubes (5 cm × 5 cm × 5 cm) made of different combinations of cementitious materials were prepared for the compressive strength test. Two water to cementitious materials ratios (0.485 and 0.4) were designed. The former *w*/cm ratio referred from the test of length change of cement mortars exposed to a sulfate solution (ASTM C1012 [33]). The later value was a typical *w*/cm ratio used in Xinjiang. A local water reducer was added at proportion of 2.2% by the mass of cementitious materials for the 0.4 *w*/cm ratio. The standard graded sand [28] was utilized with a gradation ranged in Table 4. The standard graded sand [28] was utilized. Twelve duplicated cubic specimens were molded for each type of cement mortar and tested for the compressive strength at 3, 7, 28 and 60 days. The mass of cementitious material to sand is 1 to 2.75. The detailed cement mortars molded in this test were shown in Table 5.

### 7.2. Sulfate Resistance of Cement Mortar

Sulfate corrosion in concrete usually appears in the form of expansion, cracking and spalling. Sulfate resistance of cement mortars was evaluated by measuring the length change of cement mortars immersed in a sulfate solution. According to ASTM C1012 [33], for the same mortars used in the compressive strength tests, after the compressive strength of cement mortars reached 20 MPa, the original length of mortar bars was measured and then the mortar bars were immersed in a sulfate solution. Then, the lengths of mortar bars were measured and the changes of length were calculated. A small length change indicates better sulfate resistance.

### 7.3. Permeability of Concrete

In accordance of ASTM C1202 [34], the rapid chloride permeability test was conducted to evaluate the chloride penetration of concrete. The proportions for concretes were presented in Table 6. The amount of charge passed in a certain time was used as an indicator of the permeability of concrete. It is well known that the sample age has Significant influence on test results. Most concretes, if properly cured, become progressively less permeable with time. Concrete containing SCMs needs longer time than ordinary concrete to get the same level of hydration. Therefore, in order to compare the permeability between concrete with SCMs, all the concrete specimens cut from drilled cores in field were cured for 60 days before testing. Each drilled core of each type of concrete was around 20 cm long with a diameter of 10 cm. Three duplicated specimens were cut from each drilled core and cured properly until testing. The water to cementitious materials ratios for concretes in field are ranged from 0.39 to 0.58. The sample has a 10 cm diameter and a 5 cm thickness.

## 8. Results and Discussion

### 8.1. Compressive Strength of Cement Mortar

**Figure 3.**
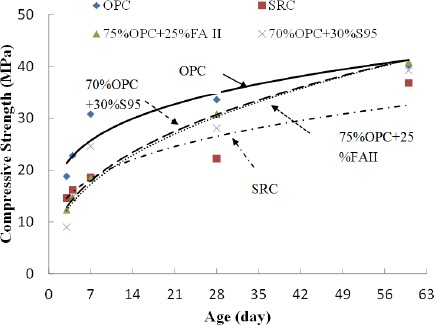
Compressive strength of cement mortars.

Figure 3 shows the compressive strength of cement mortar with 0.4 *w*/cm ratio. The 28-day compressive strength of ordinary cement mortar with 0.485 *w*/cm ratio was relatively low (15 MPa) and is not presented. The ordinary cement mortar had highest strength increase within the first 7 days of curing. SRC mortar had lowest compressive strength, maybe due to some cementitious admixtures added in it. The 28-day and 60-day results showed that cement mortars with fly ash or slag maintained same strength level as ordinary cement mortar, which could be attributed to the fact that replacing cement with fly ash can extend the process of hydration and thus allow higher strength development in the long term. It is worth to mention that the compressive strength test standards in United States and in China are different in many aspects, such as raw materials, sample size, sample preparation, curing conditions. Therefore, the compressive strength results here should not be used to grade the strength class of cements with/without SCMs.

### 8.2. Sulfate Resistance of Cement Mortar

**Figure 4.**
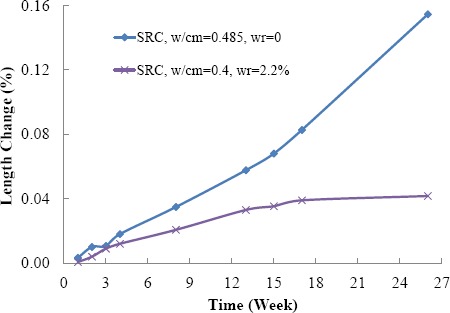
Influence of *w*/cm ratio on sulfate resistance of cement mortars.

Figure 4 shows the influence of *w*/cm ratio on sulfate resistance of cement mortar bars. It can be seen that SRC mortar with 0.485 *w*/cm ratio continuously expand in 6 months and still had high potential to expand. The SRC mortar with a 0.4 *w*/cm ratio had much less expansion and became stable after 4 months. It indicates that even with SRC, high *w*/cm ratio made concrete vulnerable under sulfate attack, which was in good agreement with the findings of other researchers [12].

**Figure 5.**
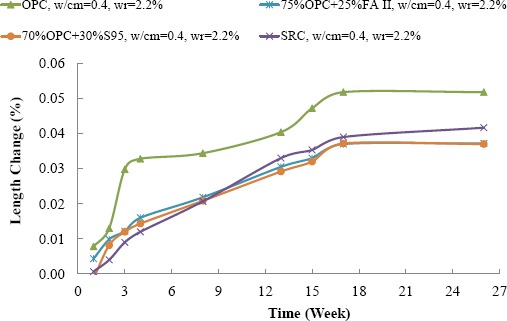
Influence of SCMs on sulfate resistance of cement mortars.

Figure 5 shows the effect of SCMs on sulfate resistance of cement mortar with 0.4 *w*/cm ratio. It can be seen that using SCMs or SRC significantly reduced the expansion of cement mortar in the sulfate solution. It is noted that the long term stability of cement mortars with SCMs were even better than that of the SRC mortar. Only the OPC mortar did not meet the criterion of American Concrete Institute (ACI) specification for sulfate resistance (C201-2R): the length change should be less than 0.05% at 6months. All other formulas satisfied the requirement and can be used in the severe sulfate environment.

### 8.3. Permeability of Concrete

**Figure 6.**
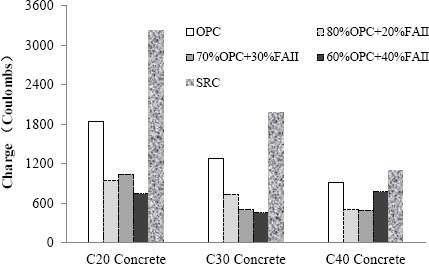
Chloride ion penetrability with varying fly ash contents.

**Figure 7.**
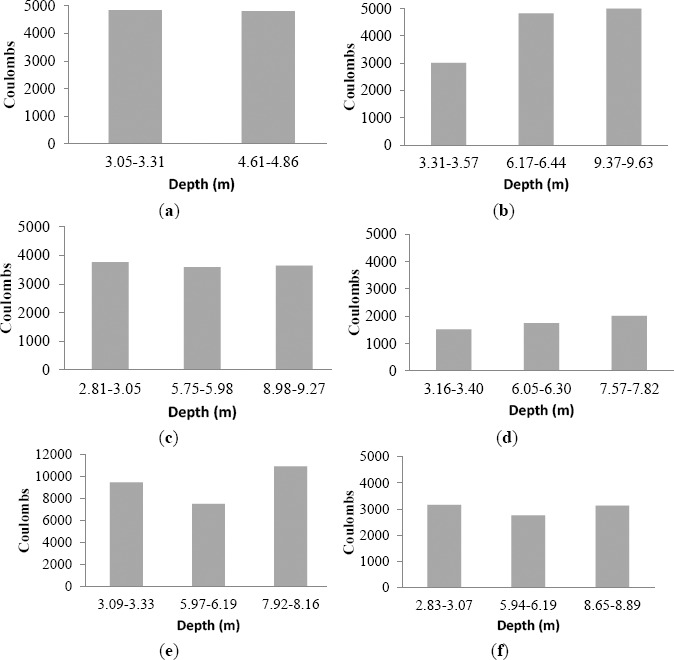
Chloride ion penetrability of C30 concrete at different depths of pile. (**a**) OPC Concrete; (**b**) 80%OPC+20%FAII Concrete; (**c**) 70%OPC+30%FAII Concrete; (**d**) 60%OPC+40%FAII Concrete; (**e**) SRC Concrete; (**f**) SRC+10%CM Admix. Concrete.

Figure 6 shows the results of chloride permeability test. It can be seen that although SRC could improve sulfate resistance of mortar, the SRC concrete was more permeable to chloride ion than ordinary cement concretes. Compared with ordinary cement concretes, adding 20%–40% fly ash reduced the chloride ion penetrability by 40%–65%. Thus, adding fly ash not only reduced expansion caused by sulfate attack but also reduced the permeability of concrete, which is beneficial to sulfate resistance. It can be seen that the chloride ion penetrability generally decreased as the strength of concrete increased due to denser microstructure. In addition, the permeability of concrete decreased with the increase in fly ash content. Figure 7 shows the results of chloride permeability of concrete cores taken at different depths of concrete pile, which also indicates that concrete permeability decreased as fly ash content increased.

Generally, concrete made with fly ash or slag not only maintained similar strength to that of ordinary cement concrete, but also had a higher sulfate resistance and a much lower permeability. Although SRC concrete had a sufficient sulfate resistance, its strength and permeability were compromised. In summary, concrete made with up to 25% fly ash or 30% slag as replacement of ordinary portland cement showed better sulfate resistance than SRC concrete and could be applied in the typical sulfate environment in Northwest China.

## 9. Conclusions

The present study investigated the chemical, mechanical, and durability properties of concrete made with local mineral admixtures and subjected to typical severe sulfate environment in Northwest China. The chemical and thermal analyses were conducted to characterize chemical components and C_3_A concentration of the cementitious materials. To evaluate the mechanical properties and durability of the cement mortar and concrete, compressive strength, sulfate resistance, and chloride permeability tests were performed. Based on the results from the laboratory tests, the conclusions can be drawn and summarized as follows:

- The chemical analysis showed that local mineral admixtures from Northwest China had higher SiO_2_ and lower Al_2_O_3_ contents than ordinary portland cement or the selected SRC, which would potentially improve the sulfate resistance of concrete by reducing permeability and reactive aluminates. The thermal analysis indicated low C_3_A content in the cement mortar with these local mineral admixtures, which was beneficial to the sulfate resistance of concrete.
- The X-ray diffraction analyses indicate that pozzolanic reactions will happen when fly ash is added into concrete, which will make concrete less permeable and more durable in sulfate and chloride environment.
- The compressive strength results showed that the cement mortars made with up to 25% fly ash or 30% slag exhibited similar compressive strength to that of ordinary cement mortar, whereas the cement mortar made with SRC had a much lower compressive strength than the former two.
- The sulfate resistance test results showed that the cement mortar made with up to 25% fly ash or 30% slag exhibited a higher sulfate resistance than the mortar made with SRC.
- The permeability test results showed that fly ash and slag significantly reduced the permeability of concrete, whereas SRC increased the permeability of concrete, putting concrete at higher risk of sulfate corrosion.

In summary, the sulfate resistance of concrete could be effectively improved by adding local mineral admixtures (class F fly ash or slag), or using the selected SRC. However, SRC significantly reduced the strength and increased the permeability of concrete; whereas use of fly ash or slag could maintain similar strength and reduce permeability concrete, which would be beneficial for the long-term durability of concrete.

## Figures and Tables

**Table 1.** Chemical analysis results of cementitious materials.

Components	SRC	OPC	Fly ash		Slag	

			FAI	FAII	S95	S75
CaO (%)	62.28	58.90	4.8	4.95	38.53	36.34
SiO_2_ (%)	21.02	22.48	59.12	57.9	34.07	37.02
Al_2_O_3_ (%)	4.00	6.43	19.19	21.26	12.42	9.85
Fe_2_O_3_ (%)	5.37	3.82	6.58	5.79	0.38	1.81
MnO (%)	0.295	0.114	0.089	0.078	0.342	1.089
MgO (%)	2.17	1.88	2.19	2.36	8.26	5.03
Na_2_O (%)	0.40	0.44	1.28	1.34	0.89	1.05
K_2_O (%)	0.52	0.57	2.56	2.75	0.69	0.91
TiO_2_ (%)	0.298	0.373	0.865	0.938	3.217	0.535
P_2_O_5_ (%)	0.20	0.08	0.19	0.25	<0.01	0.03
LOI (%)	1.96	2.06	1.52	1.21	−0.72	3.7
Total (%)	98.51	97.15	98.39	98.81	98.09	97.37

Note: LOI is the abbreviation of Loss on Ignition.

**Table 2.** Chemical compounds of two cements.

Chemical Compounds	OPC	SRC
C_3_S (%)	47	52
C_2_S (%)	22	27
C_3_A (%)	15	4
C_4_AF (%)	12	12

**Table 3.** Chemical compounds of supplementary cementing materials (SCMs).

Name	Chemical Formula	Weight (%)

S75 Slag		
Wermlandite	(Mg_7_AlFe(OH)_18_)(Ca(H_2_O)_6_(SO_4_)_2_(H_2_O)_6_)	8
Anhydrite	CaSO_4_	32
Calcite	CaCO_3_	27
Murmanite	Ti_2_Na_2_Si_2_O_9_(H_2_O_2_)	10
Tobermorite	Ca_2.25_(Si_3_O_7.5_(OH)_1.5_)(H_2_O)	10
K_2_Al_22_O_34_	K_2_Al_22_O_34_	6

S95 Slag		

Akermanite	Ca_2_Mg(Si_2_O_7_)	60
CaSO_4_·0.62H_2_O	CaSO_4_·0.62H_2_O	16
Rosenhahnite	Ca_3_Si_3_O_8_(OH)_1.9_(CO_3_)_0.1_	8
Rankinite	Ca_3_Si_2_O_7_	7
Lorenzenite	Na_2_Ti_2_Si_2_O_9_	5
Na_2_Al_22_O_34_·2H_2_O	Na_2_Al_22_O_34_·2H_2_O	4

Fly Ash I		

Quartz	SiO_2_	51
Diaoyudaoite	NaAl_11_O_17_	21
Mullite	Al_6_Si_2_O_13_	21
Na_2_Al_22_O_34_·2H_2_O	Na_2_Al_22_O_34_·2H_2_O	3
Valleriite	(Fe^2+^,Cu)_4_(Mg,Al)_3_S_4_(OH,O)_6_	2
NaAl_7_O_11_	NaAl_7_O_11_	2

Fly Ash II		

Quartz	SiO_2_	58
Diaoyudaoite	NaAl_11_O_17_	15
Mullite	Al_6_Si_2_O_13_	15
Na_2_Al_22_O_34_·2H_2_O	Na_2_Al_22_O_34_·2H_2_O	6
Tobermorite	Ca_5_Si_6_O_16_(OH)_2_·4H_2_O	4

**Table 4.** Gradation of standard graded sand [28].

Percentage passing sieves (%)					Source of sand
1.18 mm	600 μm	425 μm	300 μm	150 μm	Ottawa, IL, USA
100	96–100	65–75	20–30	0–4	

**Table 5.** Different cement mortars casted in the compressive strength tests and the sulfate bar change tests.

Types	OPC	SRC	75%OPOC + 25%FAII	70%OPC + 30%S95
*w*/cm = 0.485	×	√	×	×
*w*/cm = 0.4, water reducer = 2.2%	√	√	√	√

**Table 6.** Proportions of concretes in rapid chloride ion permeability test (RCIP) test.

Concrete Type	Cememtitious Materials	Water Cement Ratio	Water (kg/m^3^)	Cement (kg/m^3^)	Fly Ash II (kg/m^3^)	Sand (kg/m^3^)	Aggregate (kg/m^3^)
C20 Concrete	100%OPC	0.58	178	307	0	979	989
	80%OPC + 20%FAII	0.58	178	246	61	979	989
	70%OPC + 30%FAII	0.58	178	215	92	979	989
	60%OPC + 40%FAII	0.58	178	184	123	979	989
	100%SRC	0.58	178	307	0	1082	886

C30 Concrete	100%OPC	0.45	162	360	0	1033	845
	80%OPC + 20%FAII	0.45	162	288	72	939	939
	70%OPC + 30%FAII	0.45	162	252	108	939	939
	60%OPC + 40%FAII	0.45	162	216	144	939	939
	100%SRC	0.45	162	360	0	1127	751

C40 Concrete	100%OPC	0.39	163	420	0	908	909
	80%OPC + 20%FAII	0.39	163	336	84	908	909
	70%OPC + 30%FAII	0.39	163	294	126	908	909
	60%OPC + 40%FAII	0.39	163	252	168	908	909
	100%SRC	0.39	162	420	0	999	818
